# ‘Prescription’ for Purposeful Adaptation of Professionalism-and-Ethics Teaching Strategies for Remote Delivery

**DOI:** 10.3390/pharmacy9010055

**Published:** 2021-03-07

**Authors:** Cicely Roche

**Affiliations:** 1 School of Pharmacy & Pharmaceutical Sciences, Panoz Building, Trinity College, College Green, D02 F306 Dublin, Ireland; rocheci@tcd.ie; 2 Academic Practice, Trinity College, College Green, D02 F306 Dublin, Ireland

**Keywords:** remote online teaching, teaching strategies, constructivism, moral reasoning, professional identity formation

## Abstract

This case report outlines the strategies underpinning the adaptation of professionalism and ethics strand (P&E) teaching for remote delivery on a Pharmacy programme in response to COVID-19 restrictions. In line with national and University guidance, P&E teaching detailed in this report was delivered online in late 2020. Sessions were generally live and recorded, although some content was pre-recorded using video-capture software. All learning activities, recordings and supporting resources are accessible to students on the University’s Virtual Learning Environment. This report reflects on the curriculum, pedagogy and content of P&E teaching, with particular emphasis on teaching related to professional identity formation and moral reasoning competencies development. Design, development and delivery of remote online teaching is considered in the context of P&E teaching. Strategies used to plan for adaptation and delivery of interactive online teaching sessions aligned with P&E teaching are described. Key findings support a scholarship of teaching approach when planning for adaptation to remote online teaching. Purposeful consideration of existing curricular, pedagogical and instructional design enables the teacher to identify critical P&E teaching activities potentially compromised by the move to the online environment. Informed integration of available instructional tools to teaching activities follows. The report concludes with recommendations for future research.

## 1. Introduction

This case reports on the strategies underpinning the adaptation of ‘professionalism and ethics strand’ (P&E) teaching for remote delivery on a Pharmacy programme (PP) in response to COVID-19 restrictions. Curriculum design and learning outcomes for the five year PP align with accreditation requirements set out by the Pharmaceutical Society of Ireland (PSI, the Pharmacy Regulator), namely that graduates ‘will possess (a) professional and personal integrity and discipline of mind, (b) an understanding of and commitment to the ethos of professionalism and (c) the capability to … advance the practice of pharmacy and its contribution to society’ [1] (p. 2). P&E curriculum design therefore directly targets moral development of the pharmacy student and his/her understanding of the profession’s social contract with, or contribution to, society [2]. In a face-to-face classroom environment, the teacher utilises a number of core teaching activities and applied developmental tools aligned with moral reasoning and moral motivation including the Defining Issues Test (DIT2) [2,3], Intermediate Concept Measures (ICMs) [2,4] and the Professional Identity Essay (PIE) [2,5]. Details of each tool, and their purpose in developing a student’s moral reasoning competencies, professional identity and individual responsibility to the fulfilment of the profession’s social contract are as follows:

- The DIT2 is a survey for activating moral schemas used in reasoning and for assessing these schemas in terms of importance judgments in decision-making [2,3]. DIT2 scores increase following engagement with profession-specific educational interventions, [2,3] especially when constructivist teaching strategies are applied. These include repeated use of logic (e.g., use of frameworks such as the PSI Code of Conduct [6], models of professionalism [7,8] and Principlism [9]), role-play and peer interaction when engaging with dilemma scenarios [10,11,12,13]. In P&E teaching, five DIT2 dilemmas related to societal issues, each with 12 standard items, are presented, and the respondent’s task is to rate and rank the items in terms of their moral importance [14]. Teaching strategies utilise students’ engagement with DIT2 dilemmas to activate moral schemas, in order to prime students prior to peer interaction during which they further explore individual responses.
- ICMs comprise a short profession-specific ‘dilemma’ scenario, and series of action and justification options that participants rate and rank [4,10]. This approach allows bespoke development in profession-specific contexts by aligning dilemmas and options with ethical concepts of particular relevance to pharmacists [2,4,10]. Student development aligned with ICM use relates to the intermediate rather than bedrock schema level of moral reasoning competencies. Teaching strategies sequence activities in a manner that students complete the ICM individually before being assigned to groups to revisit their ranking choices and interact with peers, in a defined time-frame, to come to group ‘agreement’ regarding optimum ranking. The process of debate and negotiation towards group agreement expands the students’ range of perspectives related to the dilemma proposed.
- The PIE [5] explores how the individual understands the meaning of professionalism at a particular point in his/her development and how that understanding relates to their professional formation. This supports professional identity formation (PIF), wherein students incorporate professional values, aspirations and actions into their identity and develop increasingly complex understandings of what it means to be a professional and a member of a profession [5,7]. P&E teaching strategies engage the evolving professional in reflection and goal-setting in a process that requires active teaching in order to stimulate individual reflection and drive engagement in cycles of ‘think, share and compare’ activities with their peers [5,15].

P&E teaching delivered to first and fifth year pharmacy student cohorts prioritises moral reasoning competencies development (MRCD) [2,3,4,5,14] and professional identity formation (PIF) [5,7,8]. As the process of constructing one’s professional identity begins prior to or at entry to the PP and continues throughout professional life, intensive ‘teaching’ in professionalism and ethics is scheduled during the first 6 weeks of the PP. Core teaching strategies promote active engagement with dilemma scenarios [16,17], from societal, interprofessional and profession-specific perspectives, at multiple points in the PP. Teaching in year 5 prioritises understanding of the social contract between the profession and society, in the context of addiction pharmacy, and aims to have students enter their final 8 month experiential placement with a deeper understanding of their individual responsibility to this contract [18,19,20]. 

## 2. Professionalism and Ethics Teaching Strategies

While a blended approach to P&E teaching, including the use of online discussion boards, wikis, journals and surveys, has been previously explored [10,16,17,20,21], activities in P&E workshops and face-to-face interactive lectures generally involve paper-based surveys, one-minute papers and discussion at individual, dyad and group level. In such face-to-face sessions, the teacher (Teacher, rather than lecturer/academic, is used throughout this Case Report) can adapt choice of prompt questions and the sequence of paper-based activities to the knowledge and level of engagement demonstrated by those present. PP scheduling generally accommodates P&E sessions delivered in half-class groups of approximately 35 students. In recognition of the developmental approach adopted and PP learning outcomes, attendance at P&E sessions is compulsory and students must sign attendance sheets. It would also be apparent to the teacher if a student left a face-to-face session. 

The close proximity of engagement with small groups in a face-to-face environment, allows the teacher and students to actively respond to visual cues such as body language and facial expressions. The teacher can ‘eye-ball’ individuals to encourage hesitant students into engagement and address non-engagement by direct interaction with unwilling students. He/she can also promote interaction between students and between teacher and students by adapting to what arises during the sessions. These teaching strategies align with the PP approach to professional development. In addition, paper-based activities such as DIT2, PIE and ICM rating and ranking surveys can be completed and submitted before students leave the teaching session thereby assuring that it is the student who completes the activity him or herself.

### 2.1. The Catalyst for Rapid Change

In line with national and University guidance relating to the COVID-19 pandemic, students and teachers had to wear masks and maintain social distancing of one to two meters, including when in class, during the period addressed in this paper (October–December 2020). This effectively removed opportunity to create the preferred academic environment for P&E face-to-face teaching. Deferral of P&E engagement to a later time in the PP was neither desirable nor considered by the PP team. As a result of these considerations, teaching strategies had to adapt for 100% remote online contexts in a manner that continued to drive the type of student engagement and peer interaction that align with the PP approach to moral development in the pharmacy student. 

P&E teaching was scheduled for online delivery in late 2020. Due consideration was given to the rapid design of an optimised P&E teaching strategy for remote delivery. Reports of face-to-face to remote teaching rapid adaptation processes for P&E interactive teaching were not identified in the literature. A scholarship of teaching approach, defined as ‘a combination of reflection on experience-based knowledge and research-based knowledge on teaching literature’ [22] (p. 476), was therefore employed. A Scholarship of Teaching Model, derived from review of the literature and from experience of teaching in face-to-face, blended and virtual environments, provides a framework by which a structured approach to the process could be formulated [22] (p. 476). This model proposes that knowledge about teaching derives from a mixture of reflection on premise (curricular knowledge, why we teach the way we teach), process (pedagogical knowledge, concerned with understanding student learning) and content (instructional knowledge, concerned with strategies we use in teaching) [22] (p. 481). 

Taking premise, process and content criteria into consideration, the PP’s premise remains constant regardless of face-to-face or remote teaching interactions. The manner in which P&E teaching strategies incorporate common educational theory and principles to the process [2,5,11,14,15,23,24] have been described above. Review of P&E teaching techniques used in face-to-face interactive sessions identified challenges or potential gaps in instructional knowledge [22] and content access, which may influence adaptation to the remote delivery context. The remainder of this report explores the P&E strategies used to plan for adaptation and delivery of interactive online P&E teaching sessions in a manner that accommodates the premise, process and content considerations that underpin PP design. Findings that align scholarship of teaching literature with the approach used to adapt face-to-face P&E teaching to online delivery offers transferable insight to those engaged with professional development in pharmacy programmes. 

## 3. Adapting Interactive P&E Teaching Sessions for Remote Online Delivery

In order for constructivist theories [11,23,24,25] to be appropriately employed in online P&E teaching sessions, activities must motivate students to use logic, and to participate in role-play and peer interaction when engaging with the range of dilemma scenarios presented. Design must accommodate the absence of proximity to or visual cues from students to the teacher, and between students themselves. Session plans need to purposefully incorporate sufficient prompt questions to prime students appropriately across a range of possible circumstances [15]. Flexibility with respect to sequencing of teaching activities that stimulate individual reflection and drive engagement in cycles of ‘think, share and compare’ processes derives from having teaching resources available to the teacher in the virtual learning space during online sessions, but not visible to students unless released by the teacher. This does not necessarily align with automated or adaptive release tools promoted in learning environments such as Blackboard Learn (BBL) [26], and therefore commonly requires more detailed session-planning than when teaching face-to-face. 

When adapting teaching sessions for remote live-online P&E teaching, it is also necessary to take account of institutional and PP policy:

- Potential variation in connectivity when accessing ‘live online’ sessions remotely was accommodated by providing recordings of sessions.
- The University’s summary data protection declaration was included in all sessions.
- Student attendance at sessions referred to in this case report was compulsory and the PP team has a policy of rapidly contacting non-attenders.
- The ‘virtual’ classroom within BBL, known as Blackboard Collaborate Ultra (BBCU), was the University’s stated preferred environment for live online teaching.

Review of BBL and BBCU, in the context of the P&E instructional challenges identified, was therefore prioritised.

### 3.1. Enabling Process and Content Criteria Using Blackboard Learn (BBL) and Blackboard Collaborate Ultra (BBCU)

BBL provides access to a range of instructional tools commonly used in higher education [26] for example Discussion Boards [10,16,20]; Journals [20]; Rubrics [16,20,21]; tests and surveys [20,21], and Wikis [17,20]. Alignment of P&E activities with the grade column in BBL enables automated reminders to students to complete activities by a defined time—as occurs when developmental approaches to P&E teaching align with specific sequencing of activities. 

BBCU provides a secure virtual environment in which teachers can host webinars with students and communicate with them via voice, video, lecture slides and text chat [26]. A teacher is assured that only enrolled students, staff with access to the module and guest speakers provided with a direct link to the session, will be able to access that BBCU session. 

During a live online P&E teaching session on BBCU a teacher may, for example:

- Share files or images from within the BBCU session;
- Create polls to help clarify understanding and/or ‘force’ students to make decisions in ambiguous circumstances and/or provide students with opportunity to see how their action choices compare with their peers;
- Setup breakout rooms to which student’s may be randomly allocated or be permitted to self-assign, thereby enabling ‘share and compare’ or other teaching techniques that require peer interaction;
- Give students permission to share audio, video and chat, and to use ‘drawing tools’ on files or whiteboards shared during the session;
- Download session’ poll reports, i.e., excel spreadsheets of all attendees’ answers to all polls activated during the session, to provide a record of completion of poll activities thereby providing a prompt to the teacher to contact non-engagers in a timely manner.

As the strength of students’ connectivity is visible to the teacher during P&E sessions, teachers know when additional support may be required by a student dropping out of a session due to connectivity issues. 

While it is apparent that BBCU and BBL can provide an environment and tools to support live online sessions and related activities and resources, much of the BBL guidance to teachers aligns with designing a programme for online delivery [26]. Adaptation of P&E teaching in the PP had a different context—it involves students who had enrolled in a university in expectation of an in-person student experience, on a PP that had been designed for primarily face-to-face delivery. Conscious that prior research and practice insights related to online teaching could further inform P&E remote teaching plans, the evolving evidence base related to online teaching was also reviewed.

### 3.2. The Evolving Evidence Base Related to Adaptation of Teaching for Remote Contexts

Bearing in mind that the aim in 2020 was to support a ‘*temporary online pivot*’ [27] (p. 1), the literature highlights that temporary remote online teaching is not the same as a specialised online course and therefore evidence from online teaching literature might not directly apply [27]. Nonetheless, review of online teaching literature does ground thinking in an evidence base that underpins instructional strategies in the remote context, and reinforces commonly agreed elements of good teaching practice in all teaching environments. As previously described, the PP’s existing student-centred, outcomes focused approach to curriculum design incorporates common educational theory and principles [2,5,11,14,23,24] from which adaptation to the remote online context could proceed. Literature exploring the alignment of constructivist theory and teaching methods, especially as applied to healthcare professions’ education, provides practical advice to teachers aiming to integrate theory and practice in any learning environment [23,24]. 

The importance of planned sequencing of activities and of considering the layout of materials in a remote teaching context is prioritised in design for online delivery [28]. Linear date-order sequencing of resources and activities on a Virtual Learning Environment (VLE), rather than arranging by Topic, has been identified as more learner-friendly in the adaptation of teaching to the remote online teaching environment [27,28]. Effective online teaching depends on appropriate communication strategies including multiple avenues for synchronous and asynchronous communication, clear sign-posting to content and activities, and monitoring and supporting engagement. Online teaching has been shown to increase inclusivity objectives through, for example, the availability of recordings and captioning, and accommodation of different learner styles through incorporating both synchronous and asynchronous interactions online [28,29,30,31].

However, classroom and online environments are described as being equally complex, subtle and hard to define, and the reader is cautioned that transferring from one mode into the other is likely to be fraught with pitfalls [31]. There is agreement that online teaching sessions ought to be developed with constructivist strategies in mind, but the Manifesto for Online teaching challenges any preconception that face-to-face is necessarily ‘better’ with the statement that ‘*distance is a positive principle not a deficit*’ [30] (p. 129). The same Manifesto cautions against claims that there might be a single best practice template that can be applied to the process with the opening statement that: ‘*There are many ways to get it right online. “Best practice” neglects context*’ [30] (p. 7). This report offers insight from this ‘one’ approach to adapting interactive P&E teaching sessions for remote online delivery and in doing so, provides an account of evidence-based design and delivery in practice. 

### 3.3. Teaching Plans and Communication Strategies for P&E Interactive Online Sessions

**Figure 1:**
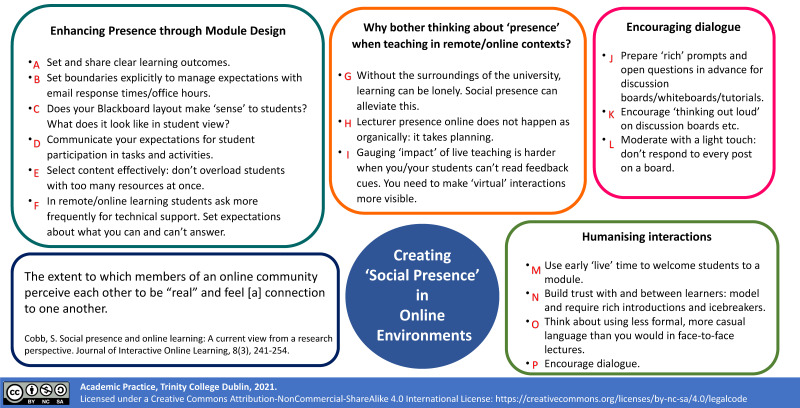
‘Creating social presence’ in live online teaching sessions: Adapted from [32].

‘Adapting our teaching for learning online’ [32,33], a module designed to support academic staff with their pedagogical practice as they prepared for remote teaching in the 2020–2021 academic year, aligns with the literature outlined above. Support for student engagement was a key priority when developing this module, and techniques to develop connections between students and teachers, as identified from the literature, are summarised in Figure 1.

As the potential for peer isolation to negatively impact on professional identity formation is a known risk in healthcare professions’ education [34], the ‘Creating ‘Social Presence’ in Online Environments’ [32,35] graphic (Figure 1), was explored in the context of P&E teaching, and used to further support planning for adaptation of P&E interactive live online teaching as outlined Table 1.

**Figure 2:**
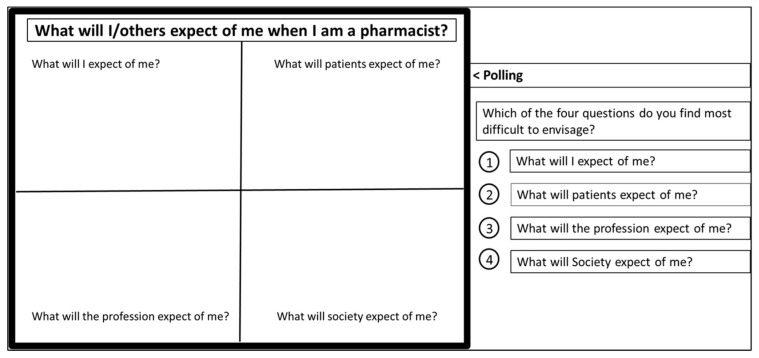
Sample use of PowerPoint slide adapted for whiteboard use and Poll tool in BBCU: Professional Identity Essay development.

The use of virtual whiteboards and polls, tools in BBCU, were identified as priority inclusions in P&E sessions. Whiteboards, with prompt questions to structure dialogue between students, and between teachers and students, were introduced either as stand-alone activities or as part of activity sequencing that included (a) using polls to prime students on an individual basis followed by (b) using breakout rooms for 3 to 7 students to share individual responses and engage in active discussion to develop an agreed group response before (c) returning to the ‘main room’ to ‘share and compare’ responses from all groups. Note that, for the reader’s convenience, a view of both a poll and a whiteboard is provided in Figure 2, but the poll and whiteboard might be presented in sequence if the process (a to c above) outlined is employed.

In a further alignment with the context [30], differing student cohort needs, i.e., at first and fifth year stages in the PP, were specifically considered.

### 3.4. Adapting Online P&E Teaching to the First Year Student Context

When undertaking P&E in their first semester in the University, students engage with a series of three two-hour workshops and four one-hour lectorials (interactive lectures), generally delivered over a six-week period [17]. Students are allocated to one of twelve groups for group activities in workshops.

Case scenarios in the three workshops (societal, interprofessional and profession-specific, respectively) are considered individually before being addressed in groups. Workshops alternate with lectorials that introduce concepts of professionalism, health policy development, and how pharmacy is practiced in primary and secondary care [17,20]. Following the P&E series students have two weeks to (a) use the wiki tool on BBL to prepare a 500-word response to an assigned question and (b) provide the group’s agreed response to a question related to the student Code of Conduct.

When teaching a session face-to-face, the teacher and peer-group members can provide practical support to students struggling to access BBL or edit the wiki. Identifying students who are struggling, at the earliest possible time in the process, is of particular urgency when students are in their first semester and at known risk of drop-out. In a deliberate effort to scaffold development of peer friendships and support, group allocations for live online teaching are aligned with those used for face-to-face laboratory classes. This approach to group allocation provides opportunity for students to get to know colleagues early in the PP. Informed by this knowledge, changes made to the online format and sequencing of activities sought to increase the opportunity to identify students at risk in a timely manner:

- BBCU poll reports were downloaded weekly to identify those not engaging.
- Individual activities were sequenced prior to live online sessions so that non completion by individual students would prompt the teacher to contact the student.
- Group activities were scheduled for completion during sessions when the teacher was available to support peer-engagement.
- Scheduling accommodated that the teacher remained in the BBCU sessions for an additional hour after each workshop, thereby scaffolding groups who choose to continue to use their breakout rooms to progress group tasks while also providing individual students with the option to visit the teacher in the main BBCU room if they had questions or concerns.

### 3.5. Adapting Online P&E Teaching to the Fifth Year Student Context

When undertaking the addiction Pharmacy module in their final semester onsite in the University, students engage with a series of seven two-hour workshops, held at weekly intervals. Workshops are delivered, in cooperation with the teacher, by exemplar practitioners (guest speakers) who include non-pharmacists as well as pharmacists [20]. The student assignment involves preparation of a video of him/herself ‘advocating’ on the topic of addiction pharmacy—usually ‘to’ the Minister of Health (Irish Government Department). Students peer review two colleagues’ draft videos during the final workshop in the series. For the purpose of this final workshop, students self-select into peer-review groups of three.

These workshops were transformed into online sessions to facilitate remote engagement. Guest speakers were invited (in May 2020) to choose whether to provide slide-decks, with speaker notes and references visible to students, in advance of the live online session or as an alternative to a live online session to provide a podcast (voiceover to run alongside the slide-deck) for upload as self-directed learning. As most of the guest speakers had never previously delivered live online sessions, and none had delivered to more than a few people at a time, the teacher assured them that they would be provided with active support preparing for and during the sessions. 

While guest speakers all committed to live online sessions, key concerns expressed by guest speakers included that interaction with students would be compromised by being remote and that, in addition to being supported by the teacher during the sessions, they would need opportunity to become familiar with BBCU in advance of scheduled teaching sessions. 

In light of these concerns, and again informed by extant literature, changes to format and sequencing of activities included that:

- Prior to each session, the teacher adapted guest speakers’ slide deck to accommodate questions for polling and prompts for virtual whiteboard activities.
- Students self-selected into one of 19 groups of three on BBL and, in order to provide a ‘visual cue’ of student presence to guest speakers, students were asked to add photos to their BBL profiles.
- The integration of peer-learning group activities to all workshops (rather than for the final workshop only) aimed to ameliorate risk of student isolation aligned with the sudden move to remote teaching. Although not all questions were used in the session, each of the 19 peer groups was required to provide a question for each guest speaker to consider prior to the session.
- The teacher opened the BBCU session prior to arrival of the guest speaker to display a ‘holding slide’ and each peer-learning group posted the group’s question related to the speakers slide-deck and core reading.
- The guest speaker integrated responses to students’ questions to an overview of their area of expertise, while the teacher managed entries to the chat, and alerted the guest speaker if students activated their microphones to engage in further dialogue.
- Student journals were used as a repository for peer review and feedback of two colleagues’ draft videos during the final workshop.

Student entries to their journals on BBL indicated that students found these sessions provided valuable opportunity to interact with guest speakers [20]. 

## 4. Findings

The use of a scholarship of teaching approach [22] to plan for adaptation of P&E teaching to the 2020 remote context provided structure and confidence to what might have been an ad hoc approach to student development. It became apparent that it was possible to demonstrate alignment of P&E adaptations with the PP’s existing curricular and pedagogical strategies, and this provided a basis on which to propose P&E teaching adaptations. The approach enabled identification of teaching strategies and activities most at risk when opportunities for face-to-face teaching were removed, and helped identify instructional tools likely to support equivalent student development in the online context. Thus, it was an informed perspective that investigated the University’s preferred VLE for potential solutions to challenges arising from the rapid pivot of P&E teaching to the online context. 

Investigation of instructional tools available in BBL and BBCU resulted in the purposeful use of polls, virtual whiteboard and peer-group activities in breakout rooms, to support ‘best practice’ [30] (p. 7) teaching in all P&E online sessions. It also enabled sequencing of activities in a manner that ensured that individual decision-making and reflection preceded and followed pair-and-share type activities throughout, as guided by the applied developmental tools aligned with moral reasoning development and professional identity formation, as detailed in Section 1 of this paper. 

The experience of delivering P&E teaching in this manner confirmed that optimised class content and the process of P&E engagement could be enabled through the strategic use of remote teaching support tools to enhance online engagement:

Polls: Timely response(s) to polls could be encouraged by identifying to students how many had yet to respond and/or that the poll was about to close. When students see the overall poll results directly after making their choice, it can prompt reflection on their own choice(s) as they compare with those of their peers—a process with known potential to directly support professional identity formation [2,5,7,34]. Student decision-making was transparent in poll results and diversity of opinion (Figure 2) frequently kickstarted active discussion on the subsequent whiteboard in a more visible manner than experienced in face-to-face teaching.

However, tight character restrictions for poll questions and the need to enter question setup in real time, limits practical use of the tool when teaching solo. While this can be accommodated by detailed planning and preparation of slide decks, there are resource implications as this is a time intensive approach. 

Whiteboards: A broad range of student entries on whiteboards was common. This enabled interactive discussion regarding topics important to the cohort, a key aim of student-centred teaching. If whiteboards were pre-arranged in a format that visually separated various prompts (e.g., Figure 2), the teacher could quickly summarise and respond to themes arising. To address the risk of an individual student feeling overlooked, an invite to students to circle anything not addressed usually prompted a couple of them to do so.

Once familiar with the use of drawing tools in BBCU, students regularly added comments or questions on white space on regular slides and this often enabled a more natural delivery style and flow, for a single teacher managing a session, than either microphone or chat formats.

Student peer-group questions in the year 5 workshop series were of such a high standard that several guest speakers used students’ questions to structure the session, rather than necessarily confining themselves to prepared slides. This enabled students to direct the session content. Group outputs from breakout room activities, when collected on a prepared slide in similar fashion, were visible to the teacher to provide immediate feedback, clarify misconceptions and/or commend insights as appropriate. 

Chat: The chat function often provided detailed nuanced questions that could be viewed during breaks in the session. Chat entries commonly included observations related to colleagues’ entries on the whiteboards. The interconnected nature of these comments and observations were unlikely to occur in face-to-face sessions.

Cameras and microphones: Students rarely turned on their cameras when in the main BBCU room, but commonly activated their microphone (rather than using the raise hand function) as a signal that they would like to join on audio. Once in the breakout rooms, cameras and microphones were used. Debriefing sessions, between the teacher and guest speakers directly after each session, generally confirmed that they found the lack of visual cues, by comparison with face to face teaching, disconcerting.

Profile pictures: Some students were reluctant to add pictures to their profiles in BBCU, even when encouraged to do so by explaining that it’s a rare visual cue available in the online space. The lack of images against students’ profiles is likely to have accentuated the disorientation experienced by guest speakers.

Breakout rooms: Students readily adapted to accessing their assigned breakout rooms, (rather than being randomly assigned), and frequently moved to and from the main room to ask questions of the teacher. Delivery of sessions using 12 to 20 breakout rooms benefitted from (a) clear instructions regarding the task, output and timeframe before they went to their rooms; (b) prior display of the slide used to capture the output from the breakout session for subsequent feedback and discussion and (c) a post-feedback whiteboard with appropriate prompt questions to capture individual learning and questions remaining following the group feedback.

While a teacher can visit individual breakout rooms, it proved to be impractical when a single teacher is engaged in large group teaching in which several rooms are activated. In addition, some students did have connectivity issues and dropped out of the session. The continued presence of a teacher in the main room provides potential to reassure students as they rejoin, and to assist access to their assigned room if needed. Teacher presence in the main room also provided all students with support if they had questions to ask. 

Timeliness of P&E activity completion, and the standard of student assignments submitted, are in line with previous cohorts. Notwithstanding that set-up time for activities delivered online can be time-consuming [27,28,29,30,31] total teaching time did not increase for year 1 students, i.e., as teaching was to the full rather than half-class, this balanced the additional time the teacher was online prior to and after scheduled sessions. Additional teaching time for year 5 was necessitated by the need to support guest speakers, but not for the two workshops directly taught by the teacher. If this approach is required in a future semester, the activity set-up will rollover to module shells established for subsequent cohorts, thereby reducing ongoing resource implications. 

## 5. Conclusions and Recommendations

Key findings support a scholarship of teaching approach to planning for adaptation of P&E teaching to remote online teaching. Purposeful consideration of existing curricular, pedagogical and instructional design, combined with review of the literature and resources aligned with teaching strategies in both face-to-face and online learning contexts, enabled the teacher to identify critical teaching activities potentially compromised by the move to the online environment. While an ideal strategy for the adaptation process was not identified in the literature, it was apparent that theoretical underpinnings common to both face-to-face and online contexts included that individual and social constructivism can be successfully designed into live online teaching [24,25,35], provided purposeful planning and extensive scaffolding are applied. Evidence also indicates that active teaching techniques and structured sequencing of individual, pair-and-share and group activities can effectively drive student development in either environment [28,29,30]. 

Integration of available instructional tools on BBL enabled teaching activities specifically identified as supportive of moral development to continue without compromising core P&E teaching strategies. The findings will thus inform planning for P&E teaching for the forthcoming semester(s). Findings may also offer some insights to those contemplating the adaptation of similar, single teacher to large group, teaching sessions in other educational contexts. 

Survey and interview of others teaching P&E could further inform developments in teaching strategies. Student surveys and focus groups could explore the student perspective of teaching activities they experience, and student performance at year-end examinations on the PP will be compared with previous cohorts.

While this case report does not claim to be a comprehensive consideration of all issues related to online teaching, findings nonetheless indicate that there are elements of the P&E teaching approach employed that might be particularly effective at driving student moral reasoning competencies development and professional identity formation in remote learning environments. Survey and/or interview of students who experience live online sessions of the type described in this case report might provide insights to what is effective for them in this context. Investigation of these elements, and of how they might be most effectively incorporated into curriculum design, could lead to improvements to face-to-face and blended PP teaching strategies, and student achievement of learning outcomes, in the future. 

## Figures and Tables

**Table 1:** Adapting teaching plans, activity sequencing and communications for P&E online sessions.

Reference A–P	Steps Taken to Adapt Teaching Plans/Sequencing of Activities and Communications for Remote Online Delivery.
**A**: Set and share clear learning outcomes	Learning outcomes included on Module home-page and in slide-decks provided.
**B**: Manage expectations with respect to response times and office hours	Students were informed that the teacher was available in BBCU from 20 min prior to scheduled start time(s); and that teacher-moderated Discussion Boards would be reviewed and responded to on a minimum of twice weekly.
**C**: Does your BB layout ‘make sense’ to students?	Activities and resources were reorganised from ‘Topic’ format to date-order sequence, and each item was released to student view at the appropriate date/time.
**D**: Communicate expectations for student participation in activities.	A summary of the P&E assignment submission dates and detailed rubrics were provided on BBL and an ‘overview’ presentation was recorded in the first session. Weekly announcements provided support. Automated reminders for completion of assignments were circulated from BBL grade centre at submission date [26].
**E**: Do not overload students with too many resources/activities at once.	Student workload, which includes workload aligned with prework for timetabled sessions and for activity completion, is defined in the PP handbook. Prework activities were transparently linked with specified session(s).
**F**: Set expectations regarding technical support.	Links to technical support services were provided in BBL and in announcements, and a video on how to use BBL/BBCU was included on the module home page.
**G**: Teacher presence online does not happen organically. It takes planning.	Detailed advance preparation of session plans included slide decks with prompt questions, formatted whitespaces, and scheduled breakout sessions. Events arising at one session frequently led to amendment of outlines for subsequent session(s).
**I**: Gauging impact of live teaching is harder … need to make ‘virtual’ interactions more visible.	Strategies employed included assuring the teacher’s camera was active during sessions; polls, prompt questions and use of whiteboards/whitespaces aligned with all sessions; ‘think-pair-share’ activities were adapted to use of BBCU breakout rooms; students were prompted to use chat, raise hand, audio, video and profile pictures.
**J**: Prepare rich prompts and open questions for discussions/whiteboards…	Whiteboards and polls (BBCU) were used extensively (e.g., Figure 2). Slide decks contained additional ‘whiteboard’ slides that could be used to adapt to specific issues arising in a given session.
**K**: Encourage ‘thinking out loud’.	Students were encouraged to use microphones, chat, and to write on slides during presentations. Slide decks included white-space for students to add their thoughts in a manner that also enabled the teacher to respond in real time (e.g., Figure 2).
**M**: Use early ‘live’ time to welcome students.	The teacher was proactive in welcoming students as they arrived. Students were encouraged to arrive early to the online session to socialise, and to ask questions if they choose.
**N**: Build trust with and between learners. Model and require rich introductions and ice-breakers.	Teacher-learner trust building activities:Early ‘live time’ informal engagement, introductions incorporated into activities, and engagement on chat, audio and whiteboards throughout live online session(s). Student-student trust building activities:Provision of clear guidelines on expected behaviours (Netiquette) in online groupwork, and small peer-group breakout room activities in all sessions. Assurance given that session recording did not begin until the scheduled start-time of the session(s).
**P**: Encourage dialogue.	Dialogue between students and teacher was encouraged by, e.g., inclusion of prompt questions and ‘think-pair-share’ activities. Dialogue between students was encouraged by using breakout rooms to complete defined activities and then share back to the main room, and the design of groupwork to include collaborative development of wiki and other group activities.

## Data Availability

Data is available from the author on request.
